# Pre-Segmented Down-Sampling Accelerates Graph Neural Network-Based 3D Object Detection in Autonomous Driving

**DOI:** 10.3390/s24051458

**Published:** 2024-02-23

**Authors:** Zhenming Liang, Yingping Huang, Yanbiao Bai

**Affiliations:** School of Optical-Electrical and Computer Engineering, University of Shanghai for Science and Technology, Shanghai 200093, China; liangzhm@163.com (Z.L.); 212190380@st.usst.edu.cn (Y.B.)

**Keywords:** 3D object detection, autonomous driving, LiDAR point cloud down-sampling, graph neural network

## Abstract

Graph neural networks (GNNs) have been proven to be an ideal approach to deal with irregular point clouds, but involve massive computations for searching neighboring points in the graph, which limits their application in large-scale LiDAR point cloud processing. Down-sampling is a straightforward and indispensable step in current GNN-based 3D detectors to reduce the computational burden of the model, but the commonly used down-sampling methods cannot distinguish the categories of the LiDAR points, which leads to an inability to effectively improve the computational efficiency of the GNN models without affecting their detection accuracy. In this paper, we propose (1) a LiDAR point cloud pre-segmented down-sampling (PSD) method that can selectively reduce background points while preserving the foreground object points during the process, greatly improving the computational efficiency of the model without affecting its 3D detection accuracy. (2) A lightweight GNN-based 3D detector that can extract point features and detect objects from the raw down-sampled LiDAR point cloud directly without any pre-transformation. We test the proposed model on the KITTI 3D Object Detection Benchmark, and the results demonstrate its effectiveness and efficiency for autonomous driving 3D object detection.

## 1. Introduction

Efficient 3D object detection in large-scale LiDAR point clouds is a crucial task for autonomous vehicles [1] to successfully recognize their environment and navigate safely, but still challenging in current situations due to the unstructured and irregular nature of the data. To address this issue, several methods have been proposed in recent years, such as (1) converting the unstructured LiDAR point cloud into regular by projection or voxelization, and then processing it with conventional convolutional neural networks [2,3,4,5,6,7,8,9,10,11,12]. However, this kind of method may cause some degree of information loss in the point cloud. (2) Designing specific methods or networks to directly act on the raw irregular LiDAR point cloud, and effectively extracting their information for feature learning [13,14,15,16,17,18,19,20].

Graph neural networks (GNNs) [21] have been proven to be a more ideal approach to deal with irregular point clouds [22,23]. This is because GNNs can effectively preserve the original features of point clouds and extract their contextual information directly, without any pre-conversions. However, the adjacent-nodes searching in GNNs involves a lot of calculations, thus limiting its application in the process of large-scale LiDAR point clouds. For 3D object detection in autonomous driving, the scale of LiDAR point clouds is commonly over hundreds of thousands, making it unfeasible to directly employ an ordinary GNN model to deal with it.

LiDAR point cloud down-sampling is a straightforward and indispensable step in current GNN-based 3D detection models in autonomous driving, but the commonly used voxel-based down-sampling (VD) or farthest point sampling (FPS) methods cannot effectively reduce the computational burden of the GNN model without affecting its detection accuracy, because they cannot distinguish the object points or background points during the down-sampling process.

In this work, we propose a novel and efficient 3D object detection model for autonomous driving, named PSD-GNN. In the model, we introduce a LiDAR point cloud pre-segmented down-sampling (PSD) method, which can coarsely pick out the object points and reduce the background points in the LiDAR point cloud frame, greatly improving the computational efficiency of the 3D detection model without affecting the model’s detection accuracy. On this basis, we build a brief graph neural network to achieve the 3D object detection goal for autonomous vehicles. The proposed method not only utilizes the advantages of the segmented-based down-sampling, that can effectively reduce the calculation burden of the model, but also utilizes the advantages of the GNN, that can extract point features from the raw LiDAR point cloud directly.

To summarize, the contributions of this work are as follows:

- We present a LiDAR point cloud pre-segmented down-sampling method, preserving the semantic information of the target objects while reducing the LiDAR point cloud scale to approximately 1/20 of the original.
- We present a lightweight GNN-based 3D detector for autonomous driving, which utilizes the pre-segmented down-sampled LiDAR point clouds as input. The proposed 3D detector achieves a competitive detection accuracy while delivering an impressive ~6× faster inference speed compared to the original GNN-based 3D detection model.
- We evaluate the proposed model on the KITTI 3D Object Detection Benchmark, and the results demonstrate its efficiency and effectiveness for autonomous driving 3D object detection.

## 2. Related Work

### 2.1. Projection-Based Methods

LiDAR-based 3D object detection is essential for an autonomous vehicle to recognize its driving environment because a LiDAR point cloud provides more accurate 3D geometry scene information than camera images. However, the point clouds generated by LiDAR are normally unstructured and irregular, so cannot be directly processed by the convolutional neural network. A straightforward idea is to convert the LiDAR point cloud into regular first, and adopt conventional CNN models to deal with it. The bird’s-eye-view (BEV) projection is a commonly used preprocessing method that uses regular-transform cognition, which involves transforming the irregular 3D point clouds into 2D image-like representations, where the *X* and *Y* coordinates of each LiDAR point are projected into a 2D grid, and the *Z* coordinate is discarded. The resulting BEV image provides a top-down view of the LiDAR point cloud, which can be processed by 2D CNNs for object detection.

BirdNet [4] and PIXOR [5] are two examples of projection-based methods that use BEV encoding for LiDAR point cloud 3D object detection. Specifically, BirdNet first projects the 3D LiDAR point clouds onto a 2D BEV cell encoding, and then uses a 2D CNN-based detector to estimate the 2D location and orientation of objects. The heights of the objects are then estimated through a series of post-processing methods, which enables 3D object detection. PIXOR uses a different approach by converting the height information of the LiDAR points into an auxiliary feature channel of the 2D BEV image, this allows the compacted BEV representation to contain both 2D and 3D information, which can be processed by conventional 2D CNNs to accomplish 3D object detection. The backbone network and the detection header in PIXOR are re-designed to estimate the categories, sizes, and orientation of objects simultaneously, which effectively improves the efficiency and detection performance of the model.

In summary, projection-based methods are convenient for applying conventional 2D CNN models to LiDAR point cloud feature learning and object detection. However, these methods can result in information loss and spatial distortion of the point clouds, which may affect the 3D detection performance of the models.

### 2.2. Voxelization-Based Methods

Voxelization is another common method of transforming LiDAR point clouds into regular, it involves dividing the 3D space into a regular grid of equally sized voxels and assigning the LiDAR points to the corresponding voxel. Once the points are assigned to voxels, they can be voxelized to a standard vector representation, which can then be processed by conventional 3D CNNs.

VoxelNet [6] is a method that divides point clouds into uniformed voxels and computes transformed voxel features by a proposed voxel feature encoding (VFE) layer. However, the massive computational cost of 3D convolutions in VoxelNet seriously affects its modeling efficiency. SECOND [7] proposes a series of improvements to VoxelNet, but the expensive 3D convolutional layers are still not replaced. PointPillars [8] is another method that applies voxelization only in the *X–Y* direction of LiDAR point clouds to form pseudo-image featurization (pillars), and predicts 3D oriented boxes for objects only using 2D convolutions. This change from 3D to 2D convolutions greatly improves the computational efficiency of the model, making the inference speed of PointPillars more than 60 FPS on a single GPU, which makes PointPillars a more efficient and practical method for LiDAR point cloud feature learning and 3D object detection tasks.

In summary, voxelization is a useful method for transforming LiDAR point clouds into a regular 3D grid of voxels which can be processed directly by conventional 3D CNN models. However, voxelization also has some limitations: it can be computationally expensive and memory-intensive, especially for large point clouds with high point densities. In addition, 3D convolutions are indispensable during the 3D voxel-vectors feature learning, hence leading to a significant increase in the computational cost of the model.

### 2.3. Point-Based Methods

PointNet [13] and PointNet++ [14] are pioneering models that can directly extract point features from the raw 3D irregular point cloud without any pre-conversion. They treat each point in the point cloud as an independent individual, and predict their category distribution through a series of nonlinear transformations and max-pooling operations.

Inspired by PointNet, point-based 3D object detection for autonomous driving has also seen vigorous development in recent years. STD [15] is a method that tries to take advantage of both voxelization-based and point-based methods; it applies a PointNet++ architecture as anbackbone to extract point features from the LiDAR point cloud, and proposes a PointsPool layer to convert the sparse point features into dense, thereby improving the computational efficiency of the model. 3DSSD [17] observes that the feature propagation layers and the proposal refinement modules in PointNet++ consume a significant portion of the inference time. To speed up the model, 3DSSD proposes a novel feature distance-based sampling strategy that effectively preserves the interior points of foreground instances. Additionally, 3DSSD designs a delicate box prediction network to substitute for the proposal refinement module in the original point-based models, which is more efficient for the estimation of objects’ 3D bounding boxes. F-PointNet [18] is a LiDAR-camera fusion-based 3D object detection method for autonomous driving which first generates a series of 2D bounding boxes of objects from the camera images, and then projects them onto LiDAR point clouds to obtain the 3D point cloud frustum proposals of the objects. Finally, two PointNet modules are applied, one for segmenting object points from the frustum proposals and the other for regressing 3D bounding boxes of objects from the segmented points. IA-SSD [19] proposes a task-oriented segmented-based LiDAR point cloud down-sampling method, which can quickly and effectively conduct non-uniform down-sampling of a LiDAR point cloud, retaining the target points belonging to the object while removing the background points as much as possible. This method helps IA-SSD achieve a good balance between the 3D detection accuracy and inference speed, allowing the performance of the point-based 3D object detection methods in autonomous driving to rise to a higher level.

In general, although point-based 3D detection methods can directly extract data features from the raw irregular LiDAR point clouds, the methodology of this kind of method regards each point in the point cloud as an isolated individual, ignoring the adjacent relationships among the points, which may affect the performance of point-based 3D detection models.

### 2.4. Graph-Based Methods

As an irregular data format, graphs can be used to represent non-Euclidean point clouds [20]. In this representation, each vertex corresponds to a point in the LiDAR point cloud, and the edges represent the relationships between points and their neighbors. By representing point clouds as graphs, graph neural networks [21] can be used on the irregular structure of a LiDAR point cloud to capture its complex spatial relationships between points, which makes GNN-based methods a promising approach for 3D object detection in autonomous driving.

Point-GNN [22] and SVGA-Net [23] are two examples of methods that use graph-based approaches for 3D object detection in LiDAR point clouds. Point-GNN reduces the scales of LiDAR point clouds by voxel down-sampling, and then, encodes the down-sampled LiDAR point cloud into a fixed-radius graph representation. For 3D object detection, Point-GNN iteratively updates the graph vertices to extract point cloud features and predicts object categories and dimensions by a graph vertex classification method in a single shot. SVGA-Net introduces the attention mechanism into the graph-based LiDAR point cloud 3D object detection, it proposes two scales of attention graphs, one for feature extraction of graph nodes and the other for feature extraction of local subgraphs. To quantify the scale of the point cloud, SVGA-Net employs an iterative FPS method to cover the original point sets. These methods showcase the potential of graph-based approaches for 3D object detection in LiDAR point clouds, and demonstrate an ongoing effort to develop more efficient and scalable graph-based methods for autonomous driving applications.

Overall, while graph-based methods can effectively extract features of non-Euclidean point clouds and have shown promise in 3D object detection applications, their computational cost remains a challenge, especially for large-scale LiDAR point clouds. This is because graph-based methods involve searching for adjacent vertices in the graph, which requires a lot of calculations.

## 3. Method

**Figure 1:**
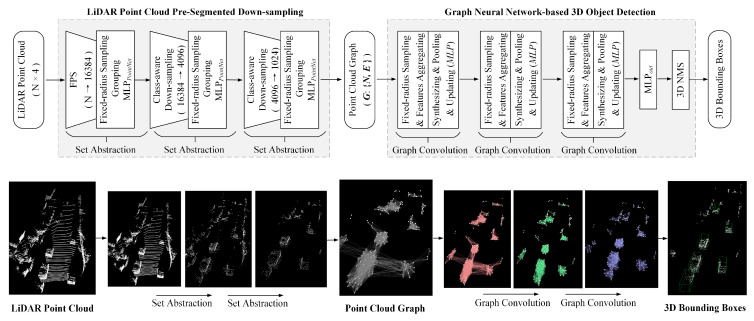
The main architecture of the proposed PSD-GNN 3D object detection model.

In this paper, we propose a novel and efficient 3D object detection model for autonomous driving, named PSD-GNN. As shown in Figure 1, the main architecture of the model consists of two components: (1) a light-weight LiDAR point cloud pre-segmented down-sampling (PSD) module; and (2) a graph neural network (GNN)-based 3D object detection module. The PSD module is designed to effectively reduce the scale of the LiDAR point cloud and ensures the semantic information of objects in the point cloud is preserved, avoiding the loss of crucial details. The GNN module is responsible for regressing 3D bounding boxes for the target objects by leveraging the graph structure of the down-sampled point clouds.

### 3.1. LiDAR Point Clouds Pre-Segmented Down-Sampling

**Figure 2:**
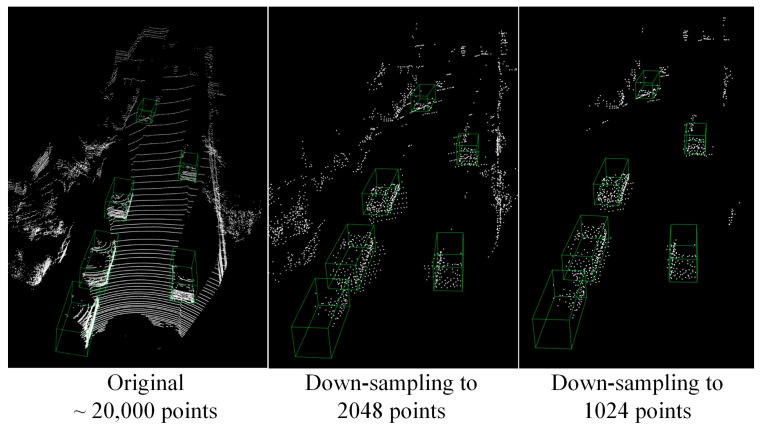
Description of the LiDAR point cloud PSD method.

As shown in Figure 2, the goal of the PSD module is to reduce the number of points in LiDAR point clouds by selectively removing background points while preserving the points belonging to foreground target objects. To achieve this goal, the PSD module adopts the concept of semantic segmentation and down-sampling, which can maintain the crucial object-related features and significantly reduce the scales of the LiDAR point clouds. By using this module, the computational cost of the following GNN-based 3D detection module will be effectively improved and its 3D detection accuracy will not be sacrificed.

**Figure 3:**
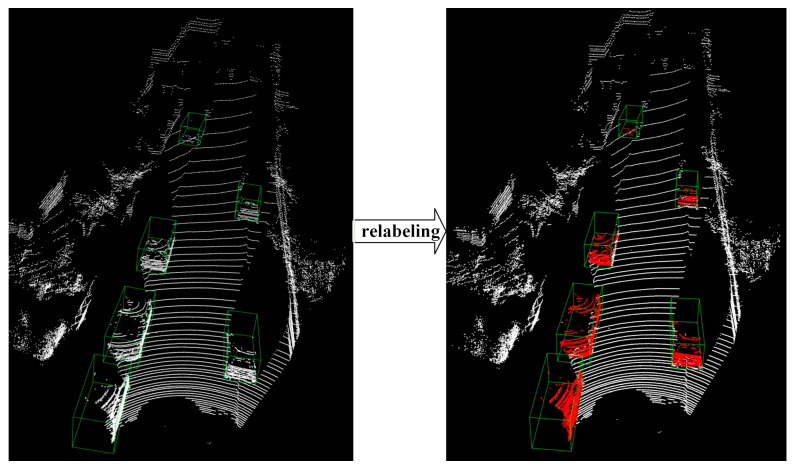
Description of the LiDAR point cloud relabeling process.

In order to enable the PSD module to distinguish between object points and background points in the LiDAR point clouds, the training data are relabeled during the training of the PSD module. This relabeling process involves generating point-wise one-hot label information for each point in the LiDAR point cloud based on the original 3D bounding box annotations of objects. As shown in Figure 3, by using the 3D bounding box annotations, the training data are relabeled in a way that each point is assigned a one-hot label indicating whether it belongs to one of the specified object classes (such as cars, pedestrians, or cyclists) or the background. In order to enable the PSD module to achieve point down-sampling, different LiDAR point cloud sampling strategies are integrated into each point semantic information learning layer. By learning the semantic information of the points in the point cloud while selectively filtering them to a smaller scale, a learning-based down-sampling strategy of the LiDAR point cloud can be achieved.

Specifically, the PSD module is implemented using a three-layer PointNet++ set abstraction (SA) layer to perform the point cloud learning-based down-sampling. In the first SA layer, we first apply a farthest point sampling (FPS) layer to rapidly reduce the number of points in the point cloud from *N* (the original number) to 16,384. To preserve the semantic information of the point clouds, the second and third SA layers employ a class-aware down-sampling (CAD) method proposed by IA-SSD [19] to reduce the number of points from 16,384 to 4096 and further down to 1024. The mathematical expression of the SA layer integrated with the down-sampling strategy can be presented as

(1) $$s_{i}^{l+1}=Sp_{down}\left\{MLP_{PointNet}\left[\sum_{N}\left((s_{j}^{l},s_{i}^{l})|d_{i,j}<r\right)\right]\right\}$$

where ***s*** represents the point features, and *i* and *j* represent the points in the point cloud. *d* represents the distance of *i* and *j*, *r* represents the fixed radius of the ball-query sampling strategy in the PointNet++ SA layer, and *N* represents the numbers of the grouping points. *Sp_down_* represents the down-sampling strategy integrated into the SA layer, and *l* represents the *l^th^* SA layer.

In detail, the first SA layer is implemented as {*N*-FPS, [radius of ball-query, *N* points of grouping, *MLP_PointNet_*]} = {16,384, [0.8, 32, [[16, 16, 32], [32, 32, 64], [96, 64]]]}. For the second and third SA layers, we set the {*N*-CAD, [radius of ball-query, *N* points of grouping, *MLP_PointNet_*]} = {4096, 1.6, 32, [[64, 64, 128], [64, 96, 128], [256, 128]]} and {1024, 4.0, 32, [[128, 256], [128, 256], [512, 256]]}. At the end, the class-aware prediction layer is set as MLP = [256, 128, 4], where 4 = cars, pedestrians, cyclists, and background.

### 3.2. Graph Neural Network for 3D Object Detection

**Figure 4:**
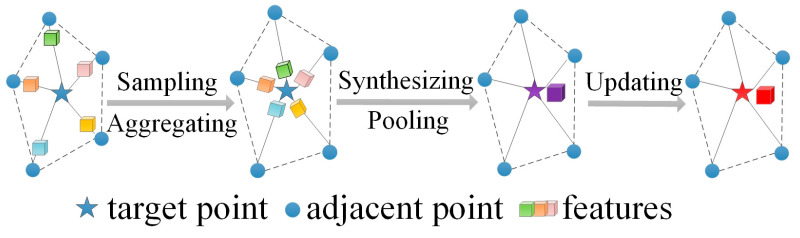
The work flow of the graph convolutional layer.

After the LiDAR point cloud is down-sampled using the PSD module, a graph neural network (GNN) composed of three graph convolutional layers is employed to detect objects in the processed LiDAR points. In the GNN’s modeling concept, each vertex corresponds to a point in the LiDAR point cloud, and the edges represent the relationships between points and their neighbors. By representing point clouds as graphs, the graph convolutional (GCN) layer has advantages in leveraging the natural geometric structure of the LiDAR point cloud and capturing spatial relationships among adjacent points. The work flow of the GCN layer can be described as shown in Figure 4: (1) a local nearest neighbor sampling operation selects the adjacent points for each point (target point) in the point cloud, and aggregates their features to the target point; (2) the aggregated features are synthesized by a ‘syn’ module (such as MLP or Conv1D) and pooled to form local information; (3) the local information of the graph is updated to a higher dimension by an ‘up’ module. The mathematical expression of the GCN layer can be presented as:

(2) $$s_{i}^{l+1}=MLP_{up}\left\{Pooling\left[MLP_{syn}\left(\sum_{(i,j)\in E}\left(s_{j}^{l},x_{j}^{l}-x_{i}^{l}\right)\right),s_{i}^{l}\right]\right\}$$

where *i* is the central point, and *j* is the nearest neighbor point of *i*. s and x, respectively, represent the point features and coordinates. $e_{i,j}^{l}=x_{j}^{l}-x_{i}^{l}$ represents the edge between point *i* and *j* inside the *l^th^* GCN layer.

In detail, the three GCN layers (sampling method, aggregating method, [*MLP_Syn_*], pooling method, [*MLP_Up_*]) in the GNN module are implemented as follows: GCN*_l-1_* = {fixed-radius ball-query sampling (*r* = 4.0 m), feature cascade, [3, 64, 128], max-pooling, [128, 256, 256]}, GCN*_l-2_* = {fixed-radius ball-query sampling (*r* = 4.0 m), feature cascade, [256, 256], max-pooling, [256, 256, 256]}, and GCN*_l-3_* = {fixed-radius ball-query sampling (*r* = 4.0 m), feature cascade, [256, 256], max-pooling, [256, 256, 256]}. To obtain the final 3D bounding box regression outputs, we employ an *MLP_out_* with units {768, 256, classes + 7 + *N*_bin_}, where classes = 4, i.e., cars, pedestrians, cyclists, and background; 7 represents the (*x*, *y*, *z*, *l*, *h*, *w*, *θ*) of the 3D box; *N*_bin_ is determined by the *MultiBin* classification method for 3D bounding box orientation estimation, and we select *N*_bin_ = 12 for this study.

### 3.3. Loss Function

In the LiDAR point cloud PSD module, cross-entropy loss is utilized to aid the module in distinguishing between object points and background points in the LiDAR point cloud. The mathematical expression of the cross-entropy loss function is presented as

(3) $$L_{cross-entropy}=-\frac{1}{N}\sum_{i=1}^{N}\sum_{j=i}^{N}\left(y_{j}^{i}log(p_{j}^{i})+(1-y_{j}^{i})\log(1-p_{j}^{i})\right)$$

where *N* denotes the numbers of object categories, $y_{j}^{i}$ and $p_{j}^{i}$ are the predicted logits, and the one-hot class label of point *i* belongs to the category *j*.

In the GNN-based 3D object detection module, different loss functions are employed for different tasks. The cross-entropy loss is used for object classification and *MultiBin*-based orientation classification, while the smooth-*l*_1_ loss function is utilized for objects’ 3D location (*x*, *y*, *z*) regression and 3D size (*w*, *h*, *l*, *θ*) regression. The expression of the smooth-*l*_1_ loss function is presented as

(4) $$smooth-l_{1}(x)=\left\{\begin{array}{cc} 0.5x^{2} & if\left|x\right|<1\\ \left|x\right|-0.5 & otherwise \end{array}\right.$$

where $x=f(x_{i})-y_{i}$, $x_{i}$, and $y_{i}$ represent the data and its annotation, and f indicates the non-linear activation function.

In addition, for precise 3D-bounding-box-size estimation, we utilize the prior mean sizes (*l*_m_, *w*_m_, *h*_m_) = [3.90 m, 1.60 m, 1.56 m], [0.80 m, 0.60 m, 1.73 m], and [1.76 m, 0.60 m, 1.73 m] of car, pedestrians, and cyclists to help the GNN module to generate more precise predictions. As expressed in (4), we parameterize the ground truth 3D box as (*x*, *y*, *z*, *l*, *h*, *w*, *θ*), and the predicted bounding box by (*x*_p_, *y*_p_, *z*_p_, *l*_p_, *h*_p_, *w*_p_, *θ*_p_). The GNN module is responsible for optimizing the residual vectors (Δ*x*, Δ*y*, Δ*z*, Δ*l*, Δ*h*, Δ*w*, Δ*θ*) between the ground truth and its predictions.

(5) $$\begin{array}{l} \Delta x=\frac{x-x_{p}}{d_{m}},\Delta y=\frac{y-y_{p}}{d_{m}},\Delta z=\frac{z-z_{p}}{h_{m}}\\ \Delta l=\log\left(\frac{l-l_{p}}{l_{m}}\right),\Delta h=\log\left(\frac{h-h_{p}}{h_{m}}\right),\Delta w=\log\left(\frac{w-w_{p}}{w_{m}}\right)\\ \Delta \theta =\sin(\theta -\theta_{p}) \end{array}$$

where $d_{m}=\sqrt{l_{m}^{2}+w_{m}^{2}}$, and Δ*θ* represents a residual rotation of the prediction angle *θ_p_* that lies within the predicted *i*^th^ bin in the *MultiBin* method.

The total loss function for the GNN-based 3D bounding box regression module can be formulated as

(6) $$L_{total}=L_{cls}+\alpha L_{loc}+\beta L_{size}+\lambda L_{\theta -bin}$$

where *L_cls_* represents the object classification loss, *L_loc_* represents the 3D location loss, *L_size_* represents the 3D size regression loss, and *L_θ-bin_* represents the *MultiBin* classification loss. Additionally, regular coefficients, *α* = 10, *β* = 10, and *λ* = 1, are introduced to adjust the proportion of each loss in the total loss function.

## 4. Experiments and Results

### 4.1. Dataset and Metrics

To certify the efficacy of our model, we conduct experiments on the renowned KITTI 3D Object Detection Benchmark. The public KITTI dataset encompasses 7481 training data and 7518 test data, which are classified into three categories: cars, pedestrians, and cyclists. We train our model on the KITTI LiDAR point cloud training set and test it on the test set.

In the test process, we adopt *mAP* (mean average precision) as our test metric. In particular, we evaluate the results for each category of object detection based on three difficulty levels: *easy*, *moderate*, and *hard*. For the ablation studies, we adopt precision, recall, and *mAP* as our evaluation metrics. The precision, recall, and *mAP* can be calculated as follows:

(7) $$\begin{array}{l} Precision=\frac{TP}{TP+FP}\\ Recall=\frac{TP}{TP+FN}\\ mAP=\frac{1}{N}\int_{0}^{1}P(R)dR \end{array}$$

where *TP* (true positive) and *FP* (false positive) represent the correct and false predictions of the positive objects, *FN* (false negative) represents the false prediction of the negative objects. *P* and *R* in the *mAP* represent the precision and recall in the *P–R* curve, respectively.

We train the LiDAR point cloud PSD module on the original KITTI LiDAR point cloud training set for 200 epochs on a single GTX 1080Ti GPU with a batch size of 4; the optimizer used in this process is Adam, with a learning rate at 0.01 and momentum at 0.9. After that, we train the GNN-based 3D object detection module on the down-sampled KITTI LiDAR point cloud training set for 1000 epochs with a batch size of 16; the optimizer used in the module is stochastic gradient descent (SGD), with an initial learning rate at 0.12 and decay of 50% every 400 epochs.

### 4.2. Quantitative Results and Discussion

To assess the performance of our model, we submitted our detection results on the KITTI LiDAR point cloud test set to the KITTI 3D Object Detection Benchmark. In Table 1, we present the quantitative 3D object detection results and compare them with those of some state-of-the-art models.

As shown in the table, our model delivers competitive performance in 3D object detection when compared with state-of-the-art (SOTA) models. Compared with the same type of graph-based model Point-GNN, we use fewer LiDAR points for training and attain better KITTI cars detection results (+0.08%, +0.71%, +1.81%). Nonetheless, our detection results for KITTI pedestrians and cyclists are slightly inferior to those of Point-GNN. This discrepancy might stem from the fact that the numbers of pedestrians and cyclists are less than the number of cars during the LiDAR point cloud down-sampling, leading the PSD module to exhibit a stronger bias toward selecting cars’ points. The inference speed of our model can achieve approximately 5 frames per second (fps) on the KITTI LiDAR point cloud dataset on a single GTX 1080Ti GPU, including LiDAR point cloud PSD speed: 15.3 ms/frame (7.6%); data IO speed: 32.6 ms/frame (16.3%); GNN model inference speed: 117.7 ms/frame (58.8%); and 3D boxes NMS speed: 34.5 ms/frame (17.3%), which is ~6× faster than the same-type (graph-based) pioneer Point-GNN model. Compared to other types of methods (voxel-based or point-based), our model achieves superior detection results primarily due to the graph convolutional network’s ability to extract topology information from raw LiDAR point clouds directly. These features can assist our 3D detection model in achieving better detection performance.

### 4.3. Quantitative Results and Discussion

**Figure 5:**
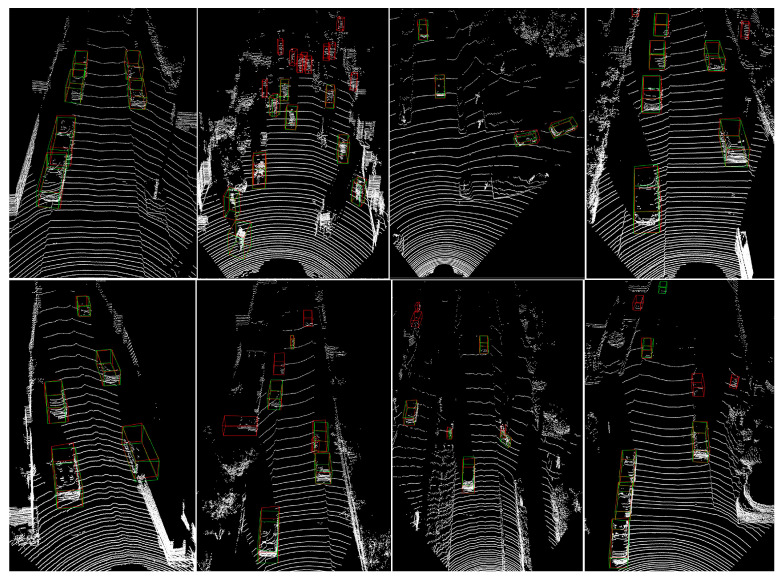
Qualitative 3D object detection results of our PSD-GNN model on KITTI LiDAR point cloud split validation subset. The ground truths and predictions are labeled as green and red, respectively.

**Figure 6:**
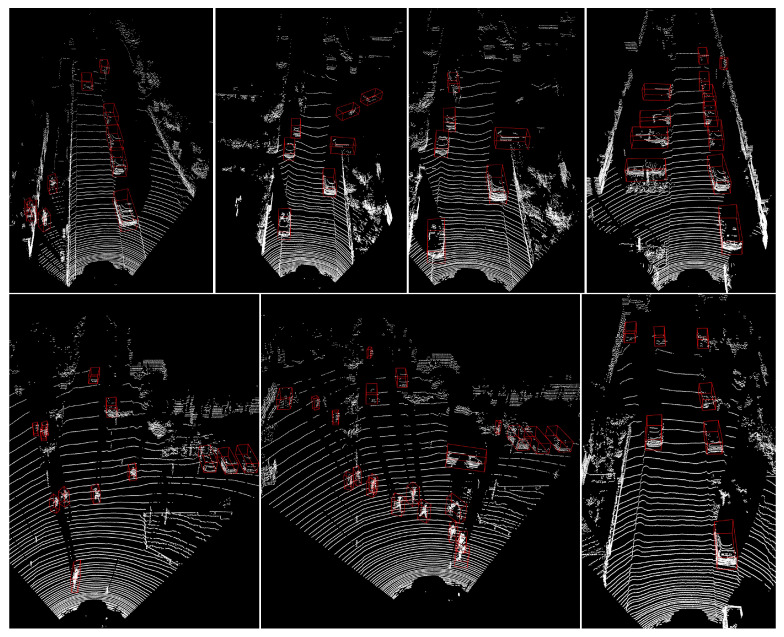
Qualitative 3D object detection results of our PSD-GNN model on KITTI LiDAR point cloud test set.

In this section, we present visualizations of our model’s 3D detection results on the KITTI LiDAR point cloud dataset to showcase its effectiveness. In Figure 5, we show the 3D detection results for cars, pedestrians, and cyclists, as well as their corresponding ground truth 3D bounding boxes on the KITTI split validation subset. Furthermore, in Figure 6, we show the detection results of our model on the official KITTI test set. The figures illustrate that our model performs well on the autonomous driving 3D object detection task, with particularly impressive KITTI cars detection results. Even for distant, unlabeled cars, our model can accurately detect them. Although our model may generate false detections for KITTI pedestrians and cyclists, we believe this issue can be mitigated through our ongoing improvements.

### 4.4. Ablation Study

#### 4.4.1. Analysis of Effectiveness of the Pre-Segmented Down-Sampling Method

In this section, we aim to evaluate the effectiveness of our proposed pre-segmented down-sampling (PSD) method in comparison to commonly used voxel down-sampling (VD) and farthest point sampling (FPS) in LiDAR point cloud-based 3D object detection methods. To achieve this, we follow the standard practice of splitting the official 7481-frame KITTI LiDAR point cloud training set into a training subset of 3712 frames and a validation subset of 3769 frames. We then train each ablation study model with the down-sampling method detailed above for 600 epochs, using the KITTI cars point clouds from the training subset, and evaluate the performance of each model on the KITTI cars point cloud validation subset. Specifically, we assess the 3D detection’s precision, recall, and *mAP* for each model.

**Figure 7:**
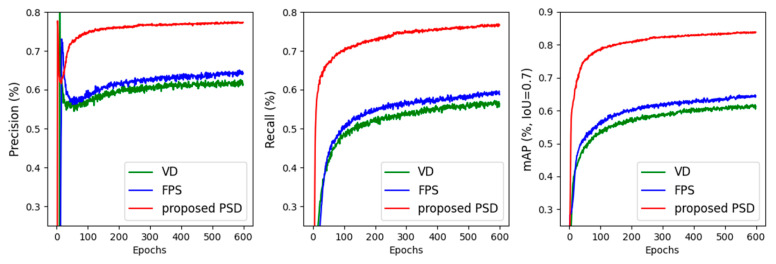
The impact of different point cloud down-sampling methods on model’s 3D detection performance.

Figure 7 provides a record of the 3D detection performance of the three models during the training process, and clearly shows that our proposed PSD method outperforms the other two methods and significantly contributes to the model’s ability to perform more accurate 3D detection. Furthermore, Table 2 presents the evaluation results and time cost of each model that employed different down-sampling strategies on the KITTI cars validation subset, which further support the effectiveness of our proposed PSD strategy in improving the performance of 3D object detection.

**Figure 8:**
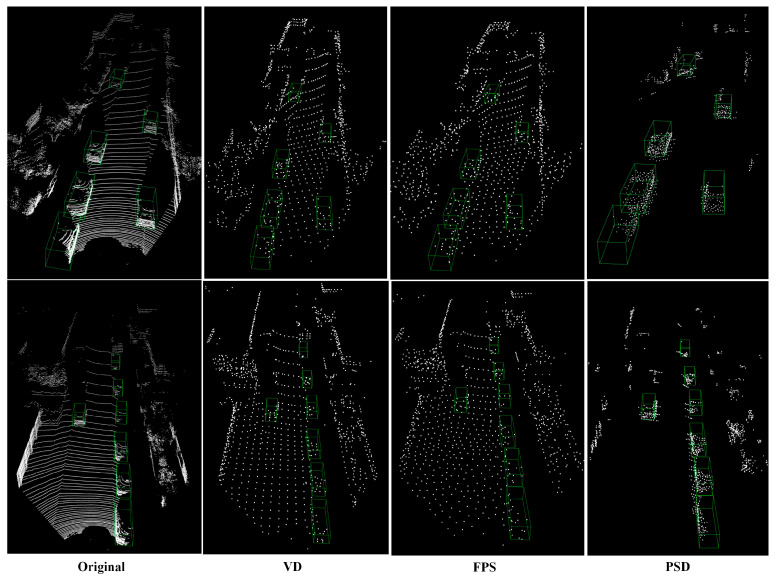
Comparative results of different LiDAR point cloud down-sampling methods.

Additionally, we provide visual evidence to demonstrate how the proposed PSD method safeguards the semantic information of the target object during the LiDAR point cloud down-sampling process. As shown in Figure 8, we uniformly down-sample the LiDAR point cloud to an equivalent scale utilizing three different down-sampling methods, proving the proposed PSD method can greatly reduce the quantity of LiDAR point clouds and ensuring the points belonging to the target object are not destroyed.

#### 4.4.2. Analysis of the Down-Sampling Scales in PSD Module

In this section, we investigate the impact of different down-sampling scales in the PSD process on the model’s 3D detection performance. We first apply the PSD method to down-sampling each KITTI cars point cloud from the training subset to three different scales: 2048, 1024, and 512 points per frame. Subsequently, we train separate models using these different point scales for 600 epochs, with respective batch sizes of 8, 16, and 32. Once the training is complete, we evaluate the 3D detection performance of each model on the corresponding scale of the KITTI cars point cloud validation subset.

**Figure 9:**
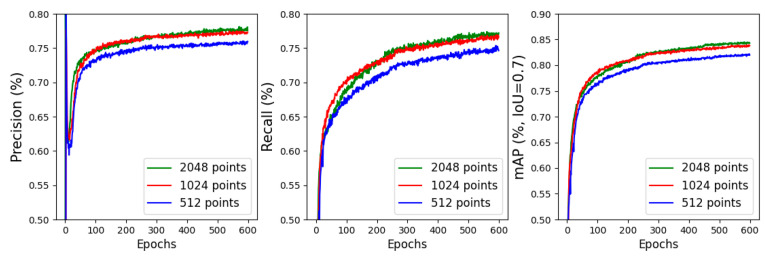
The impact of different down-sampling scales in PSD on model’s 3D detection performance.

Figure 9 provides a record of the 3D detection’s precision, recall, and *mAP* of the three models throughout the training process, and Table 3 presents the evaluation results of each model based on the different PSD module down-sampling point numbers. Based on the findings presented in the figure and table, it is evident that different down-sampling point numbers in the PSD module have varying degrees of impact on the model’s detection performance. Notably, when the point cloud is down-sampled to 1024 points per frame, there is a noticeable gap in the model’s performance change. It is important to consider the trade-off between the number of sampled data points and the computational cost of the subsequent GNN module. Increasing the number of sampled data points results in higher computational requirements, while reducing the number of sampled points can adversely affect the 3D detection performance of the model. Considering this trade-off, we have carefully evaluated the results and determined that a down-sampling scale of 1024 points per LiDAR point cloud frame strikes a balance between computational cost and detection accuracy of the model.

#### 4.4.3. Analysis of the Number of Graph Convolutional Layers in the GNN-Based 3D Detection Module

In this section, we focus on investigating the impact of different numbers of GCN layers in the GNN-based 3D object detection module on the model’s 3D detection performance. To achieve this, we conduct separate experiments for different numbers of GCN layers, specifically *N* = 1, 2, 3, and 4. For each experiment, we train a model with a specific number of GCN layers on the KITTI cars point clouds from the training subset for 600 epochs. After completing the training process, we evaluate the model’s 3D detection precision, recall, and *mAP* on the KITTI cars point cloud validation subset.

**Figure 10:**
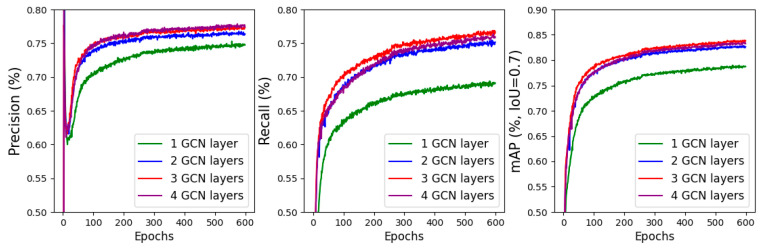
The impact of different numbers of GCN layers in GNN-based 3D detection module on model’s 3D detection performance.

Figure 10 provides a visual representation of the metric changes during the training process, while Table 4 presents the evaluation results for each model. From the observations in the figure, it becomes clear that increasing the number of GCN layers enhances the prediction ability of the GNN model. However, we notice that when the number of GCN layers reaches three, the prediction ability of the GNN model approaches convergence. Further increases in the number of layers do not significantly improve the model’s 3D detection performance. Based on these findings, we make the decision to set the number of GCN layers in the GNN-based 3D detection module to three layers.

## 5. Conclusions

3D object detection is essential for autonomous vehicles to successfully recognize their environment, and has experienced rapid development thanks to the advancements in LiDAR sensors and deep learning methods. However, the LiDAR point clouds are commonly unstructured and irregular in nature, generally requiring pre-transformations (such as voxelization or projection before they can be processed), but information loss is inevitable in these methods. Non-transformed methods, such as point-based or graph-based, may be better suited to extract point cloud information, but the heavy computational costs of these methods limit their application on low-computing-power platforms.

In order to improve the computational efficiency and better extract LiDAR point cloud features for precise 3D object detection, we propose a LiDAR point cloud pre-segmented down-sampling method in this work. This method can selectively reduce background points while preserving foreground object points in the LiDAR point cloud, thereby significantly improving the computational efficiency of the model while avoiding the loss of useful information. On this basis, we build a GNN-based 3D object detection model for autonomous driving that takes the down-sampled LiDAR point cloud as input, and directly extracts raw LiDAR point cloud features from it without any pre-transformation. We test our model on the public KITTI 3D Object Detection Benchmark, and the results demonstrate that our model not only achieves competitive detection performance but also improves the inference speed effectively compared to previous GNN models.

## Figures and Tables

**Table 1:** 3D detection performances (*AP*%) of our model on KITTI point cloud test set and comparison with other SOTA models.

Model	Reference	Category	Cars (IoU = 0.7)			Pedestrians (IoU = 0.5)			Cyclists (IoU = 0.5)		
			Easy	Mod.	Hard	Easy	Mod.	Hard	Easy	Mod.	Hard
MV3D [2]	CVPR2017	Fusion-based	71.09	62.35	55.12	--	--	--	--	--	--
PIXOR [5]	CVPR2018	Project-based	81.70	77.05	72.95	--	--	--	--	--	--
VoxelNet [6]	CVPR2018	Voxel-based	77.47	65.11	57.73	39.48	33.69	31.5	61.22	48.36	44.37
SECOND [7]	SENSORS	Voxel-based	83.13	73.66	66.20	51.07	42.56	37.29	70.51	53.85	46.90
PointPillars [8]	CVPR2019	Pillar-based	82.58	74.31	68.99	51.45	41.92	38.89	77.10	58.65	51.92
PV-RCNN [9]	CVPR2020	Point-Voxel	90.25	81.43	76.82	52.17	43.29	40.29	78.60	63.71	57.65
PV-RCNN++ [10]	IJCV2023	Voxel-Point	--	81.88	--	--	47.19	--	--	67.33	--
HVPR [11]	CVPR2021	Voxel-Point	86.38	77.92	73.04	53.47	43.96	40.64	--	--	--
TED [12]	AAAI2023	Fusion-based	91.61	85.28	80.68	55.85	49.21	46.52	88.82	74.12	66.84
STD [15]	ICCV2019	Point-based	87.95	79.71	75.09	53.29	42.47	38.35	78.69	61.59	55.30
PointRCNN [16]	CVPR2019	Point-based	85.94	75.76	68.32	49.43	41.78	38.63	73.93	59.60	53.59
3DSSD [17]	CVPR2020	Point-based	88.36	79.57	74.55	54.64	44.27	40.23	82.48	64.10	56.90
F-PointNet [18]	CVPR2018	Fusion-based	81.20	70.39	62.19	51.21	44.89	40.23	71.96	56.77	50.39
IA-SSD [19]	CVPR2022	Point-based	88.34	80.13	75.04	46.51	39.03	35.60	78.35	61.94	55.70
Point-GNN [22]	CVPR2020	Graph-based	88.33	79.47	72.29	51.92	43.77	40.14	78.60	63.48	57.08
**Ours**		Graph-based	88.41	80.18	74.10	48.09	41.16	38.71	78.13	62.45	56.14

**Table 2:** The validation results of different point cloud down-sampling methods on model’s 3D detection performance.

Method	Precision	Recall	mAP	Time Cost
VD	0.578	0.519	0.553	37.9 ms
FPS	0.601	0.542	0.596	63.1 ms
PSD	0.747	0.729	0.814	19.3 ms

**Table 3:** The validation results of different point cloud down-sampling scales in PSD on model’s 3D detection performance.

No. Points	Precision	Recall	mAP
2048	0.750	0.731	0.815
1024	0.747	0.729	0.814
512	0.722	0.705	0.783

**Table 4:** The validation results of different numbers of GCN layers in the GNN module on model’s 3D detection performance.

No. Layers	Precision	Recall	mAP
1	0.706	0.652	0.749
2	0.731	0.704	0.806
3	0.747	0.729	0.814
4	0.748	0.731	0.813

## Data Availability

The data are available from the corresponding author on reasonable request.
